# Is an increase in CD4/CD8 T-cell ratio in lymph node fine needle aspiration helpful for diagnosing Hodgkin lymphoma? A study of 85 lymph node FNAs with increased CD4/CD8 ratio

**DOI:** 10.1186/1742-6413-2-14

**Published:** 2005-09-09

**Authors:** Osvaldo Hernandez, Thaira Oweity, Sherif Ibrahim

**Affiliations:** 1 New York University Medical Center, Department of Pathology, New York, New York, USA

**Keywords:** lymph node, FNA, cytology, Hodgkin lymphoma

## Abstract

**Background:**

An elevated CD4/CD8 T-cell ratio on flow cytometry (FCM) analysis has been reported in the literature to be associated with Hodgkin lymphoma (HL). The purpose of our study was to determine the diagnostic significance of an elevated CD4/CD8 ratio in lymph node fine needle aspiration (FNA) specimens.

**Design:**

Between 1996 and 2002, out of 837 lymph node FNAs submitted for flow cytometry analysis, 85 cases showed an elevated CD4/CD8 ratio, defined as greater than or equal to 4, without definitive evidence of a lymphoproliferative disorder. The cytologic diagnoses of these 85 cases were grouped into four categories: reactive, atypical, Hodgkin lymphoma (HL), and non-Hodgkin lymphoma (NHL). Histologic follow-up was available in 17/85 (20%) of the cases.

**Results:**

5 of the 64 cases in which FCM and cytology did not reveal evidence of a lymphoproliferative disease had tissue follow-up because of persistent lymphadenopathy and high clinical suspicion. 3/5 (60%) confirmed the diagnosis of reactive lymphadenopathy. The two remaining cases (40%) were positive for lymphoma (1HL, 1NHL). 8/15 cases called atypical on cytology had histologic follow-up. 7/8 (87.5%) cases were positive for lymphoma (3HL, 4NHL). 3/4 cases called HL on cytology had tissue follow-up and all 3 (100%) confirmed the diagnosis of HL. One case diagnosed as NHL on cytology was found to be a diffuse large B-cell lymphoma. In summary, out of 17 cases with histologic follow-up 4/17 (24%) were reactive with CD4/CD8 T-cell ratio of 4.1–29, 7/17 (41%) were HLs with CD4/CD8 T-cell ratio of 5.3 – 11, and 6/17 (35%) were NHLs with CD4/CD8 T-cell ratio of 4.2 – 14.

**Conclusion:**

An elevated CD4/CD8 ratio on FCM is a nonspecific finding which may be seen in both reactive and lymphoproliferative disorders. The cytomorphologic features of the smear are more relevant than the sole flow cytometric finding of an elevated CD4/CD8 ratio.

## Introduction

Performing an excisional biopsy of an enlarged lymph node has been considered standard practice for the evaluation of lymphadenopathy, especially in-patients without prior history of lymphoma [1]. An excised lymph node provides sufficient histologic material to assess cytologic and architectural details. In many centers including ours, it is now becoming more common to forego excisional biopsy and instead perform fine-needle aspiration (FNA) as the initial work-up for lymphadenopathy [2-5]. In addition to being a safe and rapid procedure, FNAs are effective because current lymphoma classification systems place more emphasis on immunophenotype and genotype [6]. Sufficient material can be aspirated for cytomorphology, flow cytometry (FCM), and molecular studies. Even though FNAs have been shown to be effective in the evaluation of lymphomas, especially when used in conjunction with FCM, certain disorders may be difficult to diagnose.

Diagnosis of Hodgkin lymphoma by FNA biopsy is difficult [7-9]. It requires the presence of diagnostic Reed-Sternberg (R-S) cells or their variants (classic, lacunar, popcorn (lymphocytic histiocytic, L&H) and mummified) in a mixed cellular background composed of small lymphocytes, eosinophils, plasma cells, and neutrophils. The difficulty arises from the fact that the number of diagnostic cells may be limited to a few R-S cells or variants. Also, R-S cells may not be seen because the aspirated lymph node is only partially involved by the neoplastic process, which decreases the chance of obtaining the diagnostic cells, or because the neoplastic cells are hidden by fibrosis, granulomas or necrosis, which also changes the cellular background composition, and further complicates the diagnosis. Furthermore, cases were neutrophils are prominent may be mistaken for suppurative lymphadenitis [10]. In addition, this characteristic mixed cellular background is absent in cases of nodular lymphocyte predominant Hodgkin lymphoma and lymphocyte rich classical Hodgkin lymphoma, which in these conditions makes the diagnosis dependent on detection of L&H or R-S cells. On the other hand, in classical HL while the background mixed infiltrate is almost always seen, it is non-specific and may be seen in a variety of other reactive conditions.

Earlier studies examined the lymphocytic component of the background mixed infiltrates to help in diagnosing Hodgkin lymphoma and understanding the biology of this disease. Using immunohistochemical techniques the majority of lymphocytes in the background were shown to be T cells with an increase in helper/suppressor (CD4/CD8) ratio [11,12]. More recent studies showed that the mixed cellular infiltrate characteristic of HL contains an abundant amount of CD4 positiveTh2-lymphocytes [13]. Several cytokines are released by R-S cells leading to stimulation of the influx of CD4+ lymphocytes. Poppema et al. have demonstrated that CC chemokines TARC and MDC attract CD4+ T lymphocytes by binding to a CC-chemokine receptor (CCR4) found specifically on Th2 cells [14]. Fibroblasts in HL have also been shown to produce a CC-chemokine, eotaxin, which has been shown to increase the influx of Th2 lymphocytes and eosinophils [15]. This Th2 milieu generates a comfortable environment for the neoplastic R-S cells to survive and expand.

In this study we investigated the value of an increased CD4/CD8 ratio (≥4), in otherwise non-diagnostic flow cytometry findings, as a diagnostic marker for Hodgkin lymphoma in FNA biopsies. In 85 cases with increased CD4/CD8 T-cell ratio, only 7 cases were proven, using tissue sections, to be Hodgkin lymphoma with one additional case called by cytology (9%). 6 cases were proven to be non-Hodgkin lymphoma (7%). The CD4/CD8 T-cell ratio was not significantly different in these populations 5.27–13 vs. 4.2–14 with average of 7.7 vs. 7.3.

## Materials and methods

A review of the cytology records at New York University Medical Center and Bellevue Hospital revealed that between 1996 and 2002, 837 lymph node FNAs were performed in which material was submitted for FCM. In 119 cases (14%), the material submitted was insufficient for flow cytometric evaluation. Our sole selection criterion for including cases in our study was an elevated CD4/CD8 ratio by flow cytometry, defined as greater than or equal to 4. 85/718 (12%) cases fit our criteria. In all the cases, the B lymphocyte component was polyclonal and the T cells showed no aberrant immunophenotype.

All 85 aspirates were obtained on palpable lymph nodes by cytopathologists using 25 to 27 gauge needles. Aspirated material was immediately smeared on slides, air-dried, and stained with Diff-Quik and ultra-fast Papanicolaou stains. Additional material obtained for FCM was placed in RPMI solution. The cytologic diagnoses were grouped into four categories: reactive, atypical, HL, and NHL. Histologic follow-up was available in 17/85 (20%) of the cases.

Flow cytometric studies were performed using FACscan flow cytometer (Becton Dickinson, San Jose, CA). The specimens, suspended in RPMI solution, were centrifuged at 1500 rpm for 5 minutes, the supernatant was discarded, and red blood cells were lysed using Becton Dickinson FACS Lysing Solution. The cell pellets were then washed and resuspended in phosphate buffered saline. Aliquots of the cell suspension were then incubated with different combinations of monoclonal antibodies (three or four in each tube) including CD2, CD3, CD4, CD5, CD7, CD8, CD10, CD19, CD20, CD23, HLA-DR, and Kappa/Lambda light chain (all antibodies from Becton Dickinson, San Jose, CA). An average of 10000 lymphocytes was collected in the lymphocyte gate using forward and side scatter.

## Results

As shown in Table 1, the 85 lymph node aspirates were obtained from 85 patients ranging in age from 19 to 87 years. 29 patients were male and 56 were female. Eleven patients had a previous diagnosis of a lymphoproliferative disorder (1 HL, 6 mycosis fungoides, 2 NHL, 1 acute lymphoblastic leukemia, 1 Castleman disease). Lymph node sites were as follows: 14 inguinal, 43 cervical, 15 axillary, 2 epitrochlear, 5 submental, 2 subauricular, 2 intra-parotid, 1 occipital, and 1 mediastinal. The lymph nodes ranged in diameter from 0.5 to 5 cm. CD4/CD8 T cell ratios ranged from 4 to 29. A CD4/CD8 T-cell ratio of 4 was chosen as the cut-off point because it represents twice the normal value (2), and a cut-off point of 3.9 was shown to represent the lower limit of CD4/CD8 ratio is cases of HL in a recently published report [13].

**Table 1:** Summary of patient's characteristics and clinical history

Patients	No.
Total no. of FNA	85
No. of patients	85
Gender (M:F)	29:56 (1/1.9)
Age (yr.)	19–87
Site of FNA	
Cervical	43
Axillary	15
Inguinal	14
Submental	5
Epitrochlear	2
Subauricular	2
Inta-parotid	2
Occipital	1
Mediastinal	1

**Figure 1:**
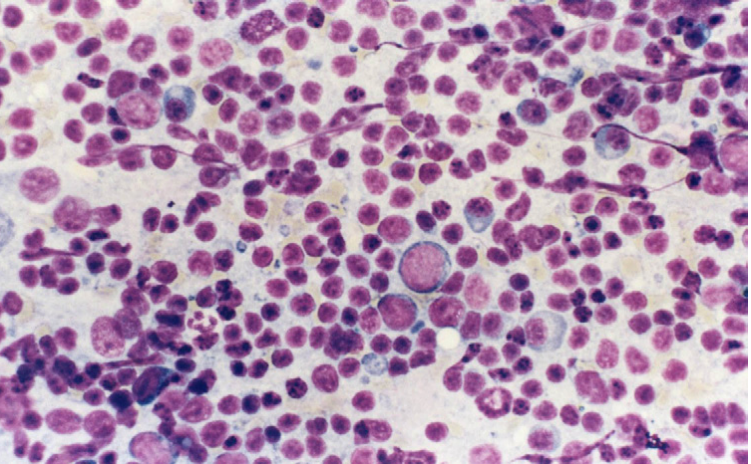
A case of reactive lymphadenopathy, cytology preparation shows a mixture of small and large lymphocytes, plasma cells and macrophages.

Overall, 64/85 (75%) lymph node aspirates were read as reactive on cytomorphology (Table 2). The cytologic findings in these cases consisted of a heterogeneous population of small and large lymphocytes with no cytologic atypicality mixed with tingible body macrophages with no definitive R-S cells or variants (Figure 1). 5 of the 64 reactive cases in which cytomorphology and FCM did not reveal evidence of a lymphoproliferative disorder had tissue follow-up because of persistent lymphadenopathy and high clinical suspicion. 3/5 cases (60%) confirmed the diagnosis of reactive lymphadenopathy.

**Table 2:** Summary of cytologic and histologic evaluation

FNA Diagnosis	# of cases (%)	Biopsy Follow up	CD4/CD8
Reactive	64 (75%)	5 (3R, 1HL, 1NHL)	4.1–29 (7.5)
Atypical	15 (18%)	8 (1R, 3HL, 4NHL)	4.6–14 (7.9)
HL	4 (5%)	3 (3HL)	5.3–13 (7.4)
NHL	2 (2%)	1 NHL	4–5.6 (4.8)

FNA: fine needle aspiration; R: reactive; HL: Hodgkin lymphoma; NHL: non-Hodgkin lymphoma. CD4/CD8 ratio range and mean.

The two remaining cases (40%) were positive for lymphoma. The first was diagnosed as nodular lymphocyte predominant Hodgkin lymphoma. Histologic evaluation revealed focal effacement of the nodal architecture by vague nodules of small lymphocytes admixed with occasional large atypical cells. Immunohistochemical stains performed on snap-frozen tissue showed that the small cells were mostly B-lymphocytes. The large atypical cells were positive for LCA, CD20 and EMA, and negative for CD15 and CD30. CD57 positive lymphocytes formed rosettes around the large cells. The second case was diagnosed as peripheral T cell lymphoma. The patient had a history of breast cancer treated by chemotherapy and radiation. The lymph node was enlarged due to an expansion of the interfollicular area by medium-sized atypical lymphocytes and increased vascularity with no significant increase in large cells. Immunohistochemistry confirmed that the atypical cells were CD3 and CD4 positive. CD8 positive T cells represented a minority of the cells. Flow cytometry failed to diagnose this case because a limited panel of antibodies against T-cell surface markers was used. The increased CD4/CD8 ratio (4.20) was thought to be a reactive change secondary to chemotherapy for breast cancer.

15 out of 85 cases were read as atypical. These cases consisted mostly of a heterogeneous population of small lymphocytes admixed with a population of intermediate or large cells, some showing angulated nuclei and prominent nucleoli with no definitive R-S cells or variants. 8/15 cases called atypical on cytology had histologic follow-up. 7/8 (87.5%) cases were positive for lymphoma (3 HL, 4 NHL). The remaining case was found to be a reactive lymph node on tissue follow-up. Three of the lymphoma cases were classical HL, nodular sclerosis type I. These lymph nodes were effaced by a mixture of small lymphocytes, plasma cells, and histiocytes forming nodules separated by collagenous bands. Within the mixed cellular infiltrate many classic R-S cells and variants, which stained strongly for CD15 and CD30, were found.

**Figure 2:**
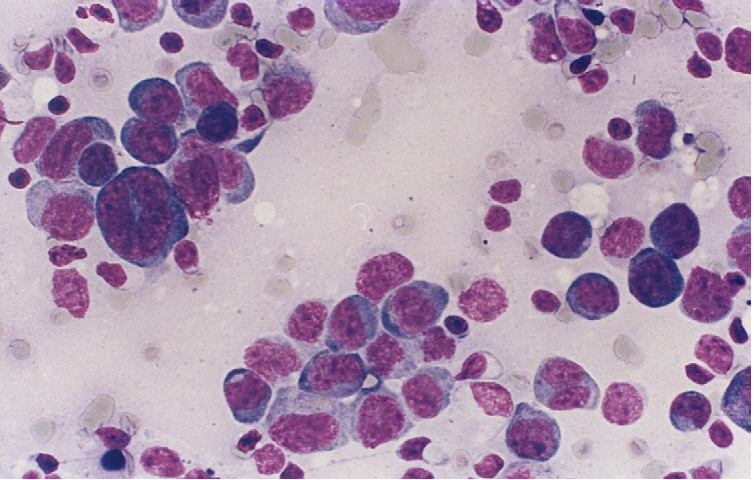
A case of diffuse large B cell lymphoma with limited amount of recovered cells and no diagnostic flow cytometry findings. The cytology preparation shows predominantly large cells with high nuclear/cytoplasmic ratio, irregular nuclei and occasional prominent nucleoli.

**Figure 3:**
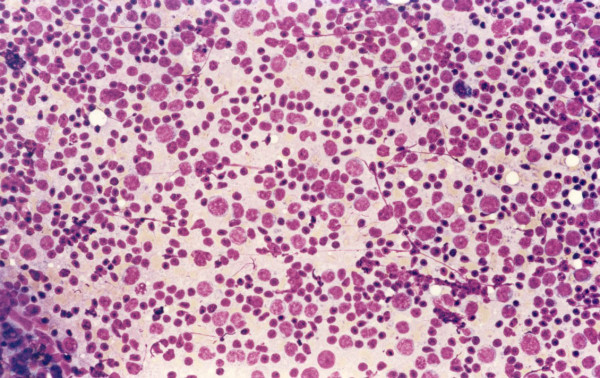
A case of follicular lymphoma, cytology preparation shows a mixture of small lymphocytes with scanty cytoplasm and irregular nuclei (centrocytes) mixed with a population of larger lymphocytes with scanty cytoplasm with rounded nuclei and single or multiple small nucleoli (centroblasts).

Two of the non-Hodgkin lymphoma cases were diagnosed as diffuse large B cell lymphomas (Figure 2). These cases show lymph nodes with sheets of large lymphocytes having vesicular nuclei and prominent nucleoli. The large cells were LCA and CD79a positive. In one case the large cells were CD30, but not CD15, positive. In these two cases flow cytometry reports noted the presence of a small population of large B cells with no surface immunoglobulin expression. A lymph node biopsy was suggested. The two remaining lymphoma cases were follicular lymphoma (Figure 3). Tissue sections showed ill-defined coalescing enlarged follicles with lost polarity, composed of sheets of large lymphocytes with no tingible body macrophages. The follicular cells were positive for B cell markers and bcl-2. In these cases flow cytometry results suggested the presence of a small population of large B cells with very dim expression of CD10 and surface immunoglobulin, called atypical and lymph node biopsy was suggested.

**Figure 4:**
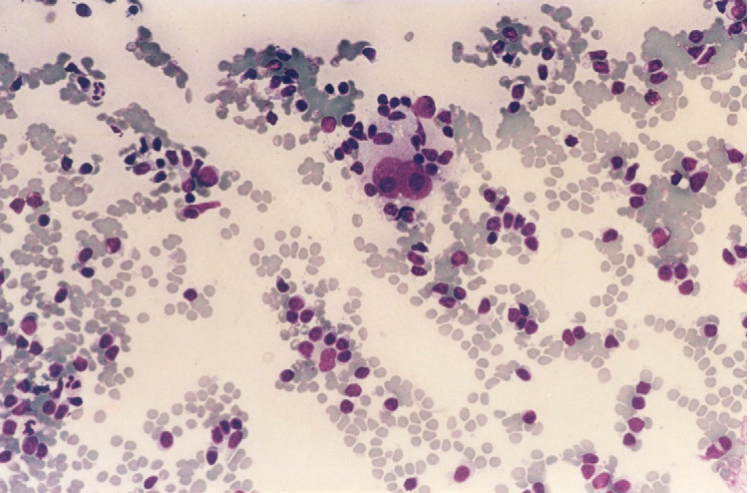
A case of classical Hodgkin lymphoma, nodular sclerosis, cytology preparation shows binucleated Reed-Sternberg cells in a mixed cellular background.

Three of 4 cases called HL on cytology had tissue follow-up and in all 3 (100%) the diagnosis of HL was confirmed. Two were classic HL, nodular sclerosis type with abundant lacunar R-S cells (Figure 4). The third case was a syncytial variant of HL, nodular sclerosis type. This case contained solid cohesive clusters of atypical cells that stained for CD30 and CD15. Flow cytometry for these three cases showed an increase in CD4/CD8 T cell ratio with polyclonal B cells.

Two of the 85 cases were diagnosed as NHL on cytology. The first case showed a population of large pleomorphic lymphocytes with significant cytologic atypia. No tissue follow-up was available for this case. The second case showed a population of large atypical lymphocytes in a background of necrosis. Tissue follow-up revealed a diffuse large B cell lymphoma. Flow cytometry study for these two cases failed to detect any large cell component and was called negative.

In summary, out of 17 cases with histologic follow-up 4/17 (24%) were reactive with CD4/CD8 ratio of 4.1–29, 7/17 (41%) were HLs with CD4/CD8 ratio of 5.3 – 11, and 6/17 (35%) were NHLs with CD4/CD8 T-cell ratio of 4.2 – 14.

## Discussion

Cytomorphology and FCM are complementary and effective in differentiating between reactive and lymphoproliferative processes, specifically non-Hodgkin lymphomas (NHLs) [3-5,16-18]. The accuracy and reliability of these techniques allows for a diagnosis without the need to perform an excisional biopsy. However, there are instances where the FCM results are nondiagnostic, but they may instead imply a certain disorder. An increased CD4/CD8 ratio in the presence of a polyclonal B lymphocyte population is one such condition. This finding has been reported in the literature to be associated with, but not diagnostic of, Hodgkin lymphoma (HL) [11-13,19,20]. Using flow cytometry, a recent study shown an increase in CD4/CD8 T-cell ratio (range 3.9 to 28 with average of 11.2) in lymph nodes of patients with classical Hodgkin lymphoma [11].

In this study, out of 85 cases read as reactive/atypical by flow cytometry, 13 were proven to be lymphoma using confirmatory biopsy (7 HD and 6 NHL). Cases of HL were not accurately diagnosed by FCM because of the paucity of neoplastic cells relative to reactive cells. CD4/CD8 T-cell ratio was not helpful in the diagnosis of HL. The average value of CD4/CD8 T-cell ratio was essentially the same for reactive, HL, and NHL cases. Overall, using the currently available antibodies, HL is not a diagnosis to be made by FCM. In cases of suspected HL, it is better to perform immunocytochemical stains on cytospin preparations or cellblock sections using the appropriate markers such as CD15 and CD30.

Six NHL cases were not accurately interpreted by FCM. One case was T-cell lymphoma and 5 cases were large B-cell lymphoma. The T-cell lymphoma case was missed by FCM because an insufficient panel of monoclonal antibodies was used and the lymphoma diagnosis was not clinically suspected. Four out of the five cases of large cell lymphoma were misinterpreted because FCM failed to confirm B-cell monoclonality. In these four cases the presence of a small population of large cells with no surface immunoglobulin restriction was mentioned and a confirmatory lymph node biopsy was suggested. Absence of immunoglobulin in these large cells is abnormal and in itself is a proof of malignancy [22]. Several recent studies highlighted the difficulty in diagnosing DLBL by flow cytometry due to lack of surface immunoglobulin expression or limited cell recovery [5,12,21,22]. These studies concluded that, in such cases diagnosis of lymphoma could be made in confidence if the cytology features and the clinical settings were reviewed.

The fifth NHL case was missed by flow cytometry because of the limited number of cells recovered from the FNA sample, which was largely necrotic. The presence of necrotic cells in FN aspiration is often an indication of malignancy. In these cases FCM may be helpful in detecting small population of clonal viable cells. However, lack of clonality does not exclude a neoplastic process and the limitation of the study has to be mentioned clearly in the FC diagnostic report.

In summary, using an increased CD4/CD8 T-cell ratio as the sole diagnostic abnormality did not help in differentiating HL from reactive or NHL cases. Cytology diagnosis and confirmation by immunohistochemical studies performed on cytospin or cellblock preparations is probably more relevant than FCM in cases of HL. In working up cases of large cell lymphoma or those with significant necrosis, communication between cytologist and hematopathologist, coordination of cytomorphology with immunophenotypic data, and knowledge of the clinical setting are essential to reach accurate diagnoses.

## Note

Corresponding article: Beaty MW, Geisinger KR: Hodgkin lymphoma flow me? Cytojournal 2005, 2:13 [23]
